# Transplantation of induced pluripotent stem cell-derived mesenchymal stem cells improved erectile dysfunction induced by cavernous nerve injury

**DOI:** 10.7150/thno.34008

**Published:** 2019-08-14

**Authors:** Zehong Chen, Xiaoyan Han, Xi Ouyang, Jiafeng Fang, Xuna Huang, Hongbo Wei

**Affiliations:** 1 Department of Gastrointestinal Surgery, The Third Affiliated Hospital of Sun Yat-sen University, Tianhe Road 600, Guangzhou, 510630, China.; 2 Central Laboratory, The Third Affiliated Hospital of Sun Yat-sen University, Tianhe Road 600, Guangzhou, 510630, China.

**Keywords:** induced pluripotent stem cell-derived mesenchymal stem cell, cavernous nerve injury, erectile dysfunction, cell therapy

## Abstract

Erectile dysfunction (ED) is an important kind of postoperative complication of pelvic surgery that affects patients' quality of life. Transplantation of mesenchymal stem cells (MSC) has been found to alleviate ED caused by cavernous nerve injury (CNI) in rats. However, little is known about whether induced pluripotent stem cell-derived mesenchymal stem cells (iMSC) have a therapeutic effect on CNI ED. We established an ED model on rats and evaluated the effect of iMSC on it.

**Methods**: Eight-week-old male Sprague-Dawley rats were assigned to four groups and received following operation: sham operation (sham group); bilateral CNI and phosphate-buffered saline (PBS) injections (PBS group); bilateral CNI and adipose-derived mesenchymal stem cells transplantation (adMSC group); or bilateral CNI and iMSC injection (iMSC group). After therapy, the cavernous nerve was stimulated by electricity and the intracavernous pressure (IAP)/mean arterial blood pressure (MAP) was measured. The endothelial and smooth muscle tissue in the penis was assessed histologically with Masson's trichrome stain. Immunofluorescence/immunohistochemical stains were applied for the detection of nNOS, vWF, eNOS, SMA, Desmin, S100β, and caspase-3. Nude rats CNI ED model was established for the evaluation of iMSC longevity and differentiation capacity. The paracrine factors were assessed by real-time PCR.

**Results**: Transplantation of iMSC significantly restored the IAP/MAP in this CNI ED model and showed long-term effects. It could rescue the expression of vWF, eNOS, SMA, and Desmin, which indicated the alleviation of endothelial and smooth muscle tissues of the penis. iMSC therapy also could increase the expression of nNOS in the cavernosum and S100β in the major pelvic ganglia (MPG) which contributed to the erectile function. Moreover, the level of BAX and caspase-3 were reduced and Bcl-2 was increased, which indicated the anti-apoptosis effects of iMSC. The iMSC showed little transdifferentiation and exerted their function by activating the secretome of the host.

**Conclusion**: Transplantation of iMSC significantly improved ED induced by CNI. The iMSC may exert their effects via paracrine factors and may be a promising therapeutic candidate for treating CNI ED in the future.

## Introduction

Defined as the insufficient ability to attain or maintain an erection for satisfactory sexual performance, erectile dysfunction (ED) is a disorder that affects physical and mental health 1. Epidemiological research has revealed that ED is becoming increasingly common among men and affected more than 300 million people in 2015 2. The erectile function of the penis requires the coordinated function of both neuronal and vascular tissues 3,4. Postoperative ED is an important kind of complication of pelvic surgery and accounts for a large proportion of ED cases. This kind of ED results from injuries to neurovascular bundles and most of these patients are unable to restore normal erectile function 5. Some preclinical studies have revealed that phosphodiesterase-5 (PDE5) inhibitors may be helpful for penile rehabilitation on postprostatectomey patients 6,7. Preliminary human trial by Schwartz et al. found that postprostatectomey patients with 100mg Sildenafil per night for 6 months showed significant increase on smooth muscle content 8. However, large clinical trials showed that the efficacy of PDE5 inhibitors was limited and other means should be explored 9-11. Thus, new treatment strategies for this kind of ED are urgently needed to make improvement on the quality of life in pelvic surgery patients.

Mesenchymal stem cells (MSC) are special cells with multi-lineage differentiation abilities 12. There is a variety of sources for MSC obtaining which covers umbilical cord blood, adipose tissue, and bone marrow 13. MSC can be amplified in vitro and used for cell-based medical therapies because of their multi-lineage differentiation potential and ability to secrete factors associated with healing 14, 15. Showing promising results, the MSC based medical therapies has been reported on some disease types including vaginal injury, corneal alloimmunity, and spinal cord injury 16-18. Recently, it was found that MSC could be potential therapeutic cells for restoring erectile function in cavernous nerve injury (CNI) related ED rat models 19. Moreover, some preliminary clinical trials used MSC in the postprostatectomy ED patients and showed positive results. For example, the human phase 1 trial conducted by Haahr et al. enrolled postprostatectomy patients suffering from ED and treated them with transplantation of autologous adipose-derived regenerative cells. After a 6 months follow up, the results showed that patients received cell transplantation showed recovered erectile function 20. The study by Yiou et al. demonstrated that intracavernous injection of autologous bone marrow-mononuclear cells was safe and effective for postprostatectomy ED patients 21. However, the procedures to obtain stem cells were all invasive and the visual analog scale for pain scoring could reach up to grade 6. Moreover, all patients need anesthesia and need to be hospitalized for stem cells collection. Last but not least, the amplification ability of MSC is low and it is easy to senescence, which significantly limit its use in clinic. Hence, it is urgently to find strategy to avoid these limitations of MSC.

Induced pluripotent stem cells (iPSC) are pluripotent stem cells generated by reprogramming somatic cells 22. iPSC can differentiate into MSC (induced pluripotent stem cell-derived mesenchymal stem cells, iMSC), providing a new source of MSC 23. The characters of iMSC includes non-invasive obtaining, easy to amplify and better homogeneity, which would be promising for circumventing the MSC drawbacks mentioned above. The optimized characteristics indicated that it could be a better idea to use iMSC rather than traditional MSC in cell therapy. Previous studies have explored the potential therapeutic effects of iMSC on some type of diseases 23, 24. For example, researchers used iMSC on chronic mouse asthma model and found that iMSC therapy exerted a long-term effect on alleviating chronic allergic airway inflammation 25. Soontararak and colleagues applied iMSC on mouse inflammatory bowel disease model and demonstrated that transplantation of iMSC helped improve intestinal health and microbiome normalization 26. However, to our knowledge, no studies have explored the effects of iMSC on CNI ED and its underlying mechanisms. In this study, we established a CNI ED model to evaluate the related therapeutic functions of iMSC. Moreover, we compared the therapeutic effects between iMSC and adipose-derived mesenchymal stem cells transplantation (adMSC). We also explored the underlying therapeutic mechanisms that iMSC might exert, hoping to have a more comprehensive understanding of its effects.

## Methods

### Human iPSC maintaining and iMSC inducing

iPSC were purchased (ATCC, Manassas, VA) and cultured in mTESR^TM^1 medium (STEMCELL Technologies, Vancouver, Canada) in six-well plates coated with Matrigel (Corning, Corning, NY). After proliferation, iPSC were passaged and cultured in knockout Dulbecco's modified Eagle's medium (DMEM; Invitrogen, Waltham, MA). The DMEM was added by 20% fetal bovine serum (FBS; Gibco, Grand Island, NJ), 1% non-essential amino acids (Invitrogen), 0.1 mM β-mercaptoethanol (Sigma, St. Louis, MO), 1mM L-glutamine (Invitrogen), and 1% penicillin-streptomycin (Gibco). After the aggregates developed, the cells were transferred to gelatin-coated plates and cultured in iMSC media (DMEM, Gibco). The media were supplemented with 10% FBS (Gibco), 1% double antibiotics (Gibco), and 2mM L-glutamine (Invitrogen). We changed the medium every 2 days and the iMSC were characterized by flow cytometry.

### Isolation and culture of adMSC

After review and discussion, this research was permitted by the Ethics Committee of The Third Affiliated Hospital of Sun Yat-sen University. Healthy young males were recruited to donate human adipose tissue. We obtained the written informed consent from the participators whose tissues were included in this study. Human adMSC were isolated using a previously reported protocol 27. Human adMSC were resuspended and seeded in culture medium containing low-glucose DMEM (Gibco) supplemented with 10% FBS (Gibco) and 1% double antibiotics (Gibco). We placed the cultures in a humidified incubator at 37°C with 5% CO_2_. Trypsin/ethylenediaminetetraacetic acid was used for passage when the cells reached 75% confluence.

### Characterization of MSC

We characterized iMSC and adMSC by incubating them in specific-conjugated antibodies. First, the nonspecific antigens of the cells were blocked with 1% bovine serum albumin (Gibco). Then the cells were subjected to the following specific conjugated antibodies: CD11b, CD29, CD31, CD34, CD44, CD45, CD73, CD90, CD105, and HLA-DR. Phosphate-buffered saline (PBS; Gibco) were used to wash the cells two times. We evaluated the expression of specific markers by FACSCalibur flow cytometer and Cell Quest software (BD Bioscience).

### Real-time PCR

TRIzol reagent kit (Invitrogen) was applied for the extraction of total RNAs from penile tissue. Reverse transcription of RNA was conducted with the PrimeScript^®^ RT reagent Kit (TaKaRa). For classical PCR, we adopt Premix Taq (TaKaRa) on GeneAmp PCR System 9700 (Applied Biosystems) using specific human or rat primers and made electrophoresis using 1% agarose. The primers are showed in Table S1. For quantitative real time PCR, SYBR^®^ Premix Ex Taq™ (Perfect Real Time) (TaKaRa) and ABI PRISM 7000 sequence detector (Applied Biosystems) were used. The GAPDH gene was used as an internal control. The primers used are presented in Table S2.

### PKH67 for cell labelling

The cells were digested and washed by no serum medium three times. Then the Diluent C (Sigma-Aldrich, St. Louis, MO, USA) were added to the cells to make cell suspension. PKH67 ethanolic dye solution (Sigma-Aldrich) were added to the above cell suspension and mixed well to disperse. The cell suspension were incubated for 2 mins in room temperature and stopped by equal volume of fetal bovine serum. We centrifuged the cells and washed the cell pellet with complete medium and put to use. The cells were injected into the cavernosum of CNI ED nude rat. The PKH67 labelled cells were detected by laser scanning confocal microscope (CarlZeiss LSM710, Oberkochen, Germany) in 3 and 7 days after cell administration.

### Animal treatment

Eight-week-old Sprague-Dawley rats with an average weight of 250 g were purchased from Guangdong Medical Laboratory Animal Center (Guangzhou, China). Eight-week-old nude rats with an average weight of 250 g were obtained from Charles River (Beijing, China). The rats were kept in an animal center and allowed to acclimate to the surroundings. The food and water were abundant and easy to obtain by the rats. The Institutional Animal Care and Use Subcommittee of The Third Affiliated Hospital of Sun Yat-sen University has reviewed and permitted the present study.

The rats were anesthetized with 2.5-3% isoflurane before surgery. A 4-5 cm incision in middle hypogastric region was made to expose the major pelvic ganglia (MPG) and the cavernous nerve (CN). CNI was performed (CNI group) and other rats receive laparotomy without CNI (sham group). We used a non-serrated hemostat (Karl Storz) to crush bilateral CNs in the CNI group. Each CN was crushed by hemostat in the 1 mm location away from MPG for 120 s. The CNI rats were assigned to 3 subgroups, which received the following: (1) an injection of adMSC (20 μL PBS containing 1×10^6^ adMSC cells; adMSC groups); (2) an injection of iMSC (20 μL PBS containing 1×10^6^ iMSC cells; iMSC group); or (3) an injection of 20 μL PBS (PBS groups). In accordance with the manufacturer's instructions, a Porcine Fibrin Sealant Kit (Hangzhou Puji Medical Technology Development Co., Ltd.) was applied for preparing cell fibrin scaffolds. We inserted the needle into the lateral cavernosum about 5 mm, withdrawed the needle slowly and made injection simultaneously.

### Evaluation of erectile function

Four weeks after treatment, the SD rats were subjected to functional evaluation and sample collection. Three months (90 days) after MSC injection, the nude rats received erectile function evaluation and sample collection. The CN was electrically stimulated to evaluate erectile function. The rats were anesthetized with 2.5-3% isoflurane. For the detection of mean arterial pressure (MAP), we carved from the up chest to the neck and showed right carotid artery. The MAP was measured by heparinized 24-gauge silastic cannula. To expose the corpus cavernosa for measurement of intracavernous pressure (ICP), we stripped off the skin of the penis. We implanted a detective needle on the root of penis. Using the two-way electrode (1.5 mA/20 Hz), we excited the CN at for 5/6 min. We recorded maximal ICP (ICPmax) and total ICP during tumescence. Pressure curves for MAP and ICP were recorded by BL-420s Biological Functional System (Chengdu Taimeng Technology Ltd.). Erection function was determined using the ratios of ICPmax and total ICP to MAP. After erectile function was evaluated, the penis and the MPG were collect for further assessment.

### Masson's trichrome staining

A portion of the penis was cut and washed with PBS. The 4% paraform was used for fixation and paraffin for embedding. This thickness of the slice was 5μm and prepared using Masson's trichrome staining. With this stain, the corpus cavernosum smooth muscle cells appear red while the collagen fibrils appear blue. The tissue sections were photographed using a digital camera and three sections per rat were selected for statistical analysis. Quantitative image analysis was made using Image-Pro Plus 6.0 software (Media Cybernetics, Rockville, MD).

### Immunofluorescence and immunohistochemical staining

The remaining portion of the penis and the MPG segments were collected. The tissues were prepared as 10μm frozen slices and properly preserved until use. For the purpose of detecting fluorescence, we fixed the penis and MPG sections in methyl alcohol for 15 min at 4°C. PBS was used to wash the sections and the mixed liquor (bovine serum albumin and Triton) were used for blocking. The penis samples were covered by antibodies nNOS (Abcam; 1:200), eNOS (Abcam; 1:100), RECA-1 (Abcam; 1:50), SMA (Abcam; 1:200) and Desmin (Abcam; 1:100). The MPG slices were bathed with S100β (Chemicon; 1:100) and caspase-3 (Abcam; 1:200). PBS was used for washing the sections and the conjugated antibodies for daylight 488 or 555 (Invitrogen) were adopted for incubation. Nuclei were stained by DAPI. We then used a fluorescence microscope to visualize signals and obtain digital images. Image-Pro Plus 6.0 software (Media Cybernetics) was applied to assess the degree of fluorescence.

For immunohistochemical staining, the penile tissue was prepared as 4 μm slices. The samples were then rehydrated, received antigen retrieval, and blocked by non-immune serum for 30 min. The samples have incubations with antibodies, including vWF (Abclonal; 1:200) and nNOS (Abcam; 1:200). We detected immunoreactions using the Cytomation Envision HRP System (Dako) and redye the sections with hematoxylin (Sigma). We performed quantitative image analysis using Image-Pro Plus 6.0 software.

### Western blot

Proteins were extracted from the penis with the RIPA protein extraction reagent (Beyotime, Beijing, China). The extraction reagent was supplemented with phenylmethylsulfonyl fluoride (Beyotime). The BCA Protein Assay Kit (Beyotime, Beijing, China) was obtained for detecting protein concentrations and 40 μgaliquots of protein were subjected for electrophoresis. After electrophoresis, the protein was passed on to PVDF membrane and probed with primary antibodies including nNOS (Santa Cruz Biotechnology; 1:500), caspase-3 (Abcam; 1:1000), Bcl-2 (Cell Signaling Technology; 1:1000), BAX (Cell Signaling Technology; 1:1000) and β-Actin (Abcam; 1:500). We bathed membranes with secondary antibody (Abcam; 1:5000) and visualized the bands using enhanced chemiluminescence substrates (Millipore, MA).

### Statistical analysis

GraphPad Prism V.7.0 (GraphPad, La Jolla, CA) was used for the comparisons between groups and statistical analysis. We presented the data are means ± standard deviations. The differences among groups were compared using analysis of variance and Newman-Keuls post hoc analysis. P<0.05 was set as statistical difference.

## Results

### Characterization of iMSC and adMSC

**Figure 1:**
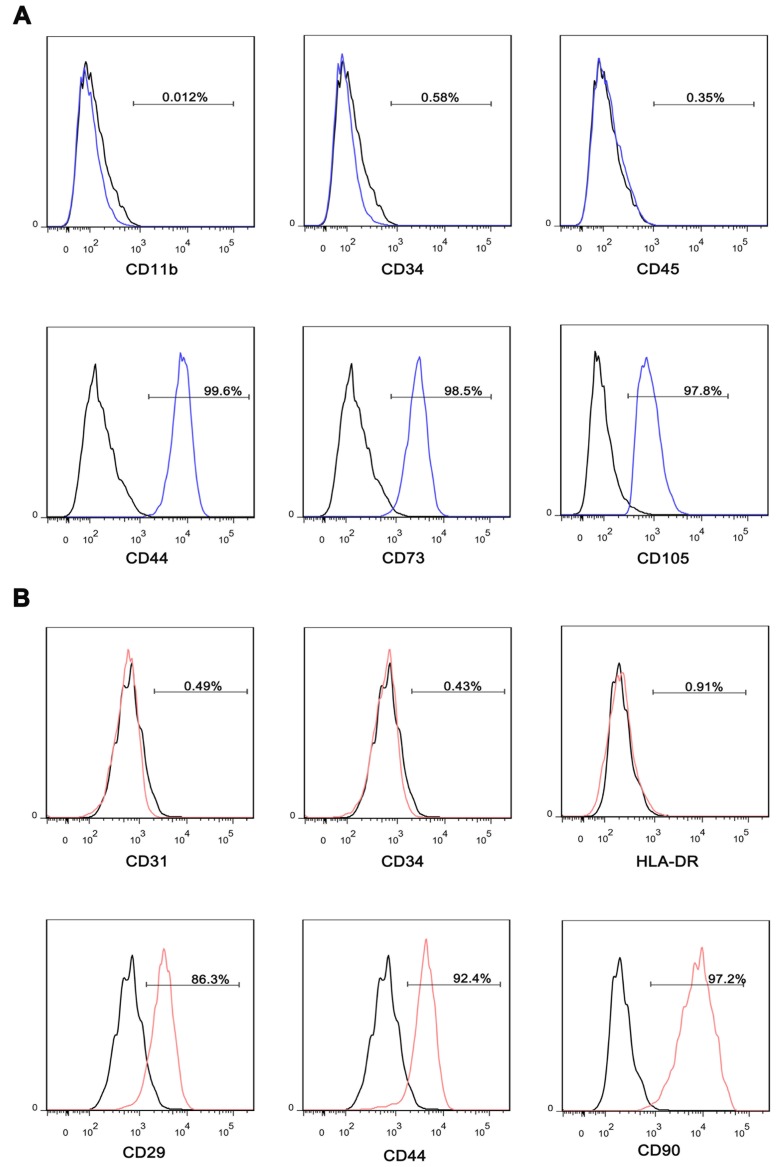
Characterization of iMSC and adMSC. (A) Flow cytometric analysis of surface markers suggested that iMSC were positive for well-known MSC markers, including CD44, CD73, and CD105, but not for endothelial or hematopoietic markers CD11b, CD34, and CD45. (B) Flow cytometric analysis of surface markers indicated that adMSC exhibited CD29, CD44, and CD90 but not for CD31, CD34, and HLA-DR.

iMSC were induced from iPSC, and adMSC were isolated from healthy volunteer's adipose tissue. After induction or isolation, the iMSC and adMSC were identified by specific surface antigens using flow cytometry. The iMSC exhibited well-known MSC markers, including CD44, CD73, and CD105, but not endothelial or hematopoietic markers CD11b, CD34, and CD45 (Figure 1A). The adMSC expressed CD29, CD44, and CD90, but not CD31, CD34, and HLA-DR (Figure 1B).

### iMSC therapy improved erectile function in CNI rat models

**Figure 2:**
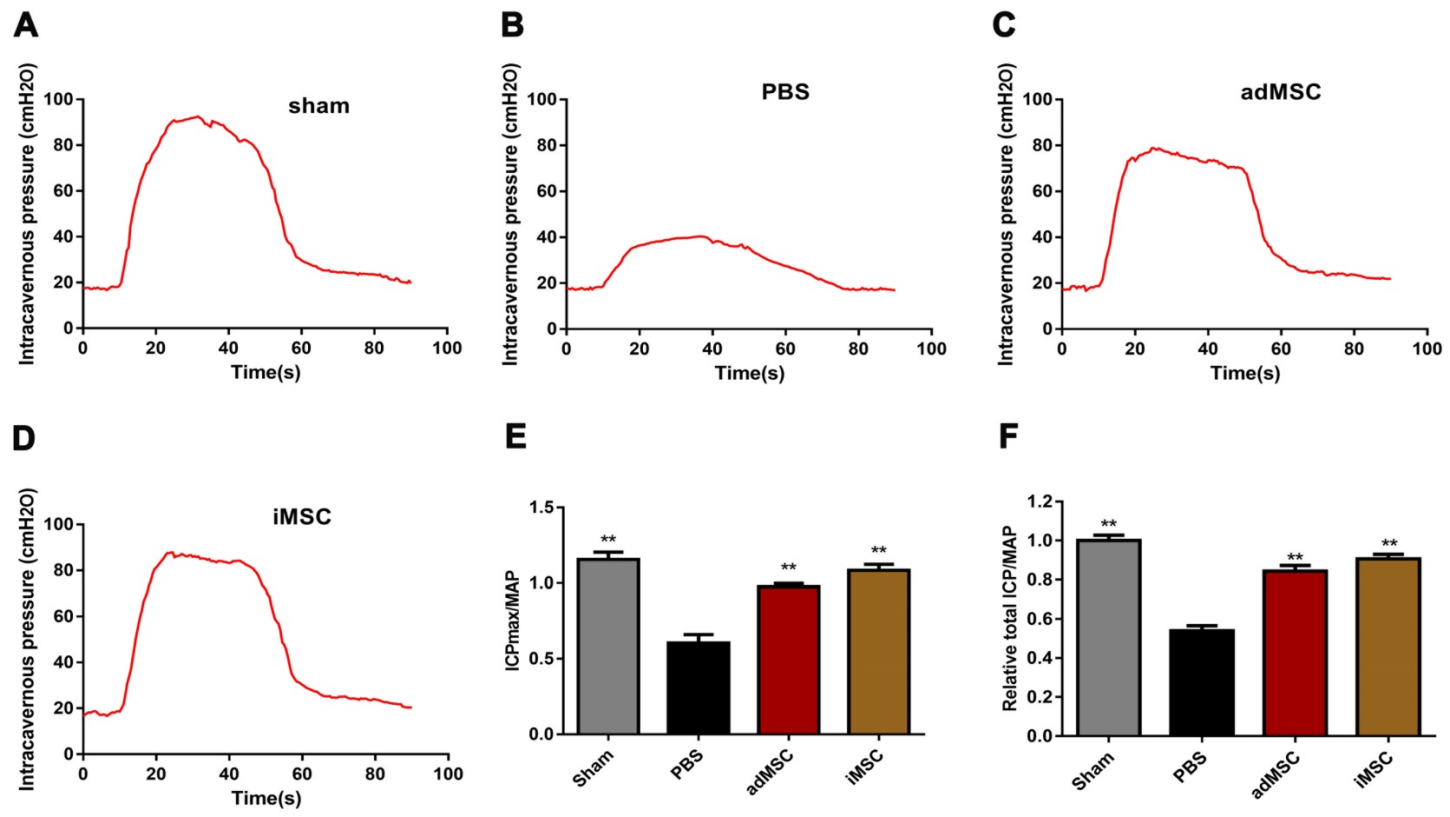
Transplantation of iMSC improved erection function of CNI rats. (A-D) ICP responses to electrostimulation in the sham, PBS, adMSC, and iMSC groups. (E) Maximum ICP to MAP ratio responses to electrostimulation in the four groups. (F) Total ICP to MAP ratio responses to electrostimulation in the four groups. Error bars: mean ± SD. **p<0.01 comparison with PBS group.

We established CNI rat models and evaluated whether injection of iMSC or adMSC could exert any effect on the injury. Four weeks after treatment, the CN was electrically stimulated and blood pressure changes (MAP and ICP) were recorded. There was no difference in MAP among four groups during electrostimulation. However, the maximal ICP (ICPmax)/MAP and total ICP/MAP were remarkably decreased in PBS group than sham group (Figure 2A-B). In addition, transplantation of iMSC or adMSC significantly improved the ICPmax/MAP and total ICP/MAP ratios when compared with the PBS-treated group (Figure 2C-D). The administration of iMSC or adMSC induced noteworthy recovery of erectile function at 4 weeks after treatment (Figure 2E-F). However, the markers of erectile function did not differ significantly between the groups treats with iMSC and adMSC.

### iMSC therapy restored the endothelial ingredient and smooth muscle ingredient in penile

**Figure 3:**
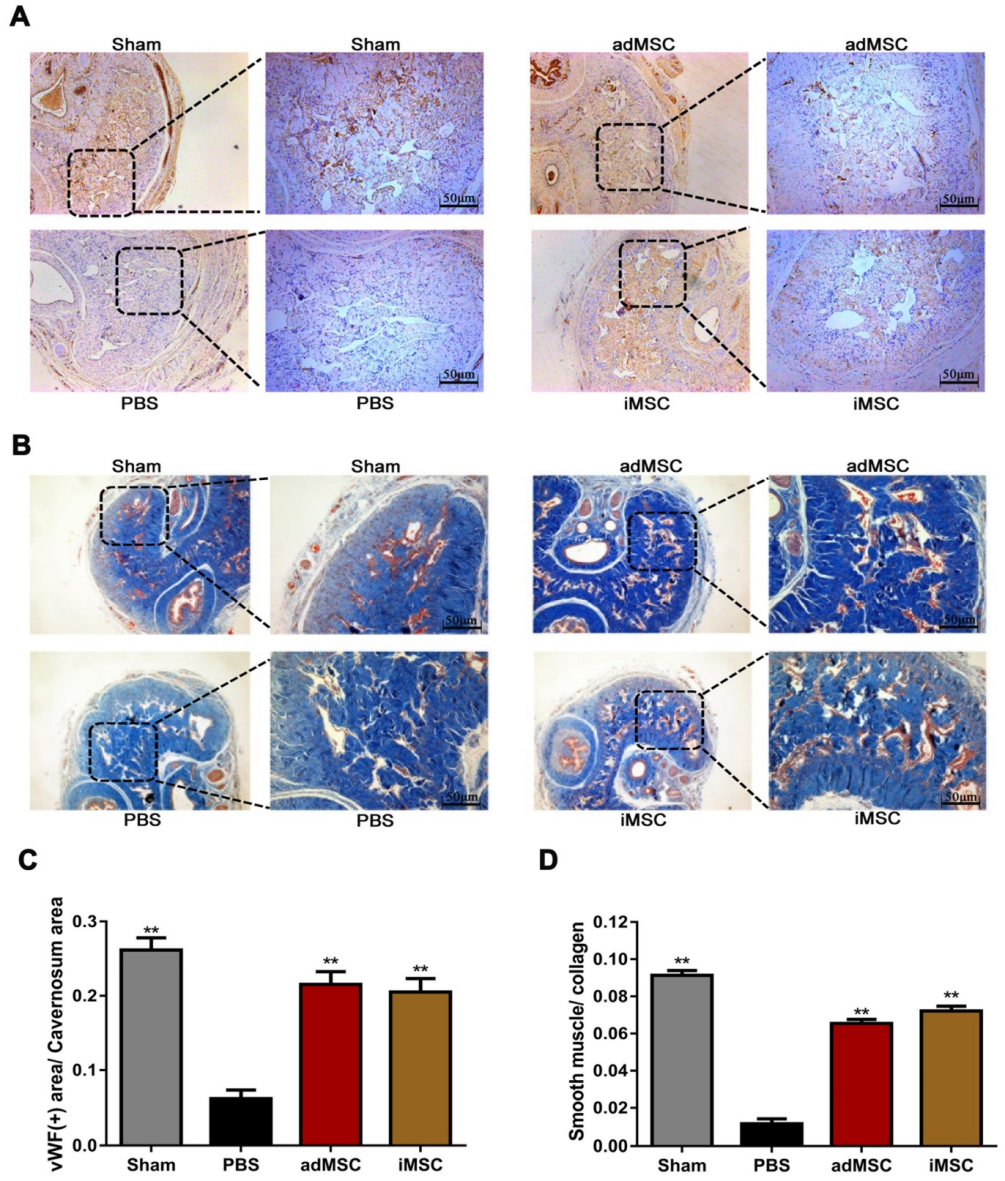
iMSC therapy restored the endothelial and smooth muscle ingredient of penile. (A, C) Immunohistochemical staining of vWF in the penile of sham, PBS, adMSC, and iMSC groups. (B, D) Masson's trichrome staining of the penile used to assess the smooth muscle/collagen ratio in the four groups. Error bars: mean ± SD. **p<0.01 comparison with PBS group.

**Figure 4:**
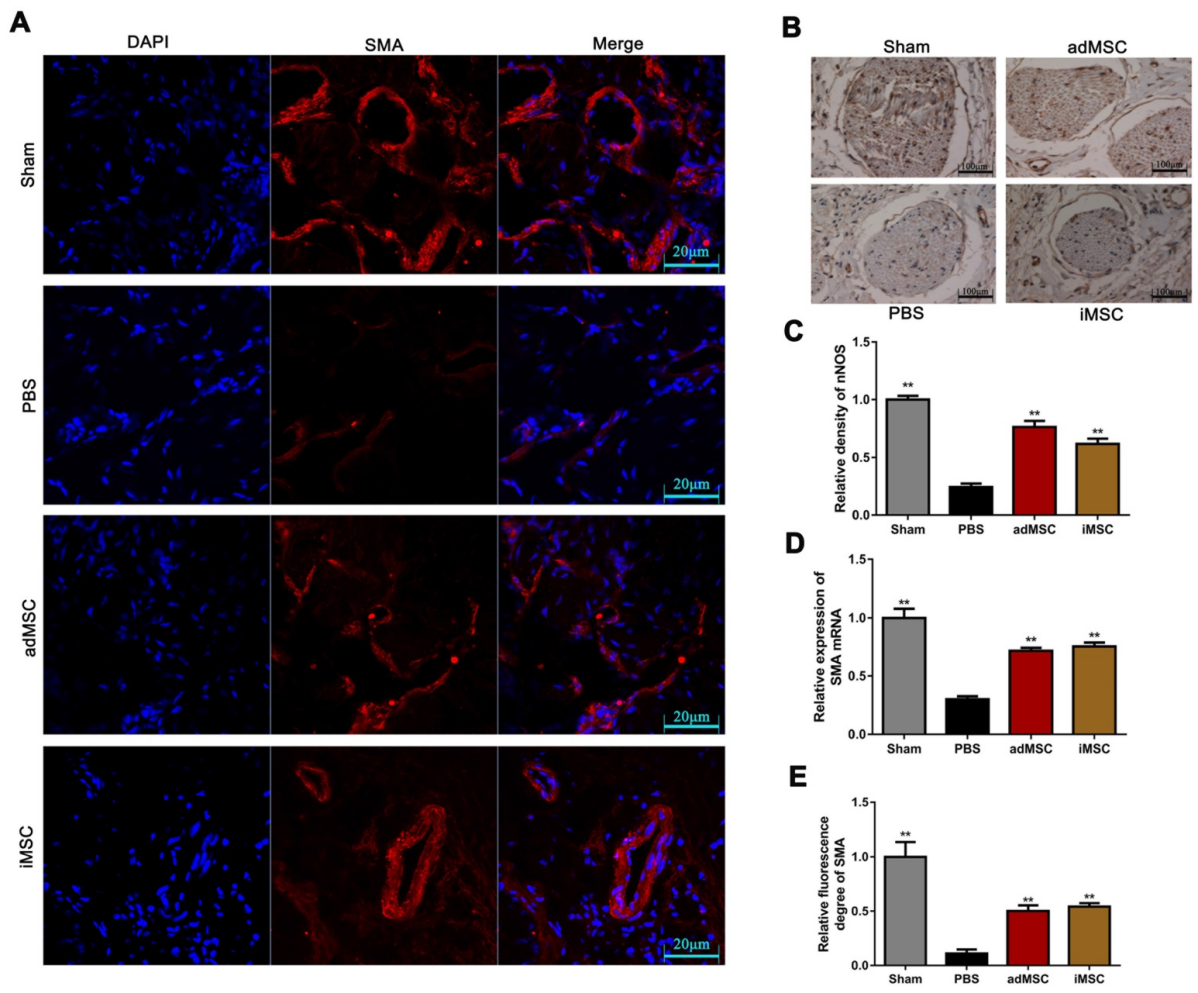
iMSC therapy increased SMA expression in the penis and nNOS in the dorsal penile nerve. (A, E) Immunofluorescent staining of SMA in the penis in sham, PBS, adMSC, and iMSC groups. (B-C) The level of nNOS in dorsal penile nerve was evaluated by immunohistochemical staining in the four groups. (D) Detection of SMA RNA by real-time PCR in the four groups. Error bars: mean ± SD. **p<0.01 comparison with PBS group.

The rats were then received euthanasia and the penile tissues were collected for further analysis. The endothelial and smooth muscle tissues of the penile are closely related to erection function and sexual performance. The expression of vWF in the penis tissues was evaluated by immunohistochemical staining, which showed that CNI can induce notable downregulation of vWF. Moreover, treatment with iMSC or adMSC significantly restored vWF expression, indicating the recovery of endothelial components in penile tissues (Figure 3A&C). The detection of another important endothelial marker, eNOS, showed consistent results (Figure S1A&C). Masson's trichrome staining was applied to assess the ratio of smooth muscle to collagen. This ratio was markedly lower in the PBS group than in the sham group, yet rescued in the iMSC and adMSC groups (Figure 3B&D). Moreover, the level of a smooth muscle cell marker, alpha smooth muscle actin (SMA), was determined by immunofluorescent staining in four groups. Consistent with the former results, iMSC or adMSC therapy was notably able to attenuate the decrease in SMA expression caused by CNI (Figure 4A&D-E). The evaluation of Desmin, which was another important marker of smooth muscle cells, showed similar results (Figure S1B&D). Taken together, these data indicate that iMSC are as effective as adMSC in restoring the endothelial ingredient and smooth muscle ingredient of the penile.

### iMSC therapy increased nNOS expression and penile tissue weight

**Figure 5:**
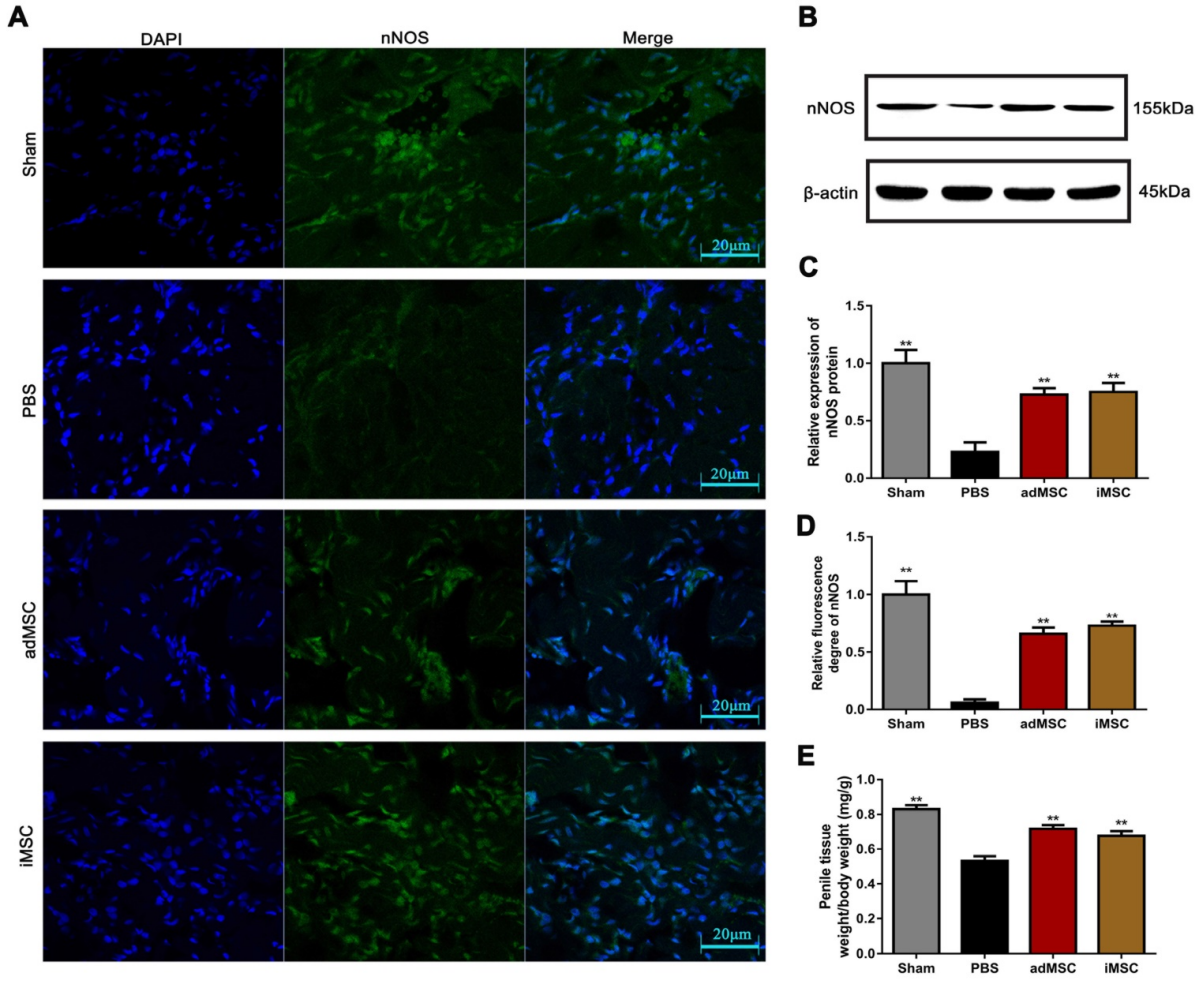
iMSC therapy increased nNOS expression and the weight of the penis. (A, D) Immunofluorescentstaining of nNOS in penile tissue from the sham, PBS, adMSC, and iMSC groups. (B-C) nNOS protein expression in penile tissues from the four groups was determined by western blot. (E) Penile tissue weight to body weight ratios were evaluated in the four groups. Error bars: mean ± SD. *p<0.05, **p<0.01 comparison with PBS group.

Immunofluorescence and immunohistochemical staining assays were applied to determine the expression of nNOS in penile tissues. As shown, the intensity of nNOS fluorescence in the penile was obviously lower in the PBS group than in the sham group, but the decrease could be profoundly mitigated by iMSC or adMSC therapy (Figure 5A&D). Similarly, the immunohistochemical staining of the dorsal penile nerve revealed that iMSC were equivalent to adMSC in restoring nNOS expression in the penis after CNI (Figure 4B-C). Furthermore, western blot revealed that nNOS protein expression was higher in the iMSC group than PBS group (Figure 5B-C). In addition, all the penile tissues and rats were weighed at 4 weeks after treatment. The penis tissue weight to body weight ratios were remarkably lower in the PBS group than the sham group, which indicated penile atrophy. However, penile weight to body weight ratios were higher in the iMSC and adMSC groups than PBS group, suggesting a therapeutic effect from the stem cell injections (Figure 5E).

### iMSC therapy exerted anti-apoptotic effects and rescued S100β expression in the MPG

**Figure 6:**
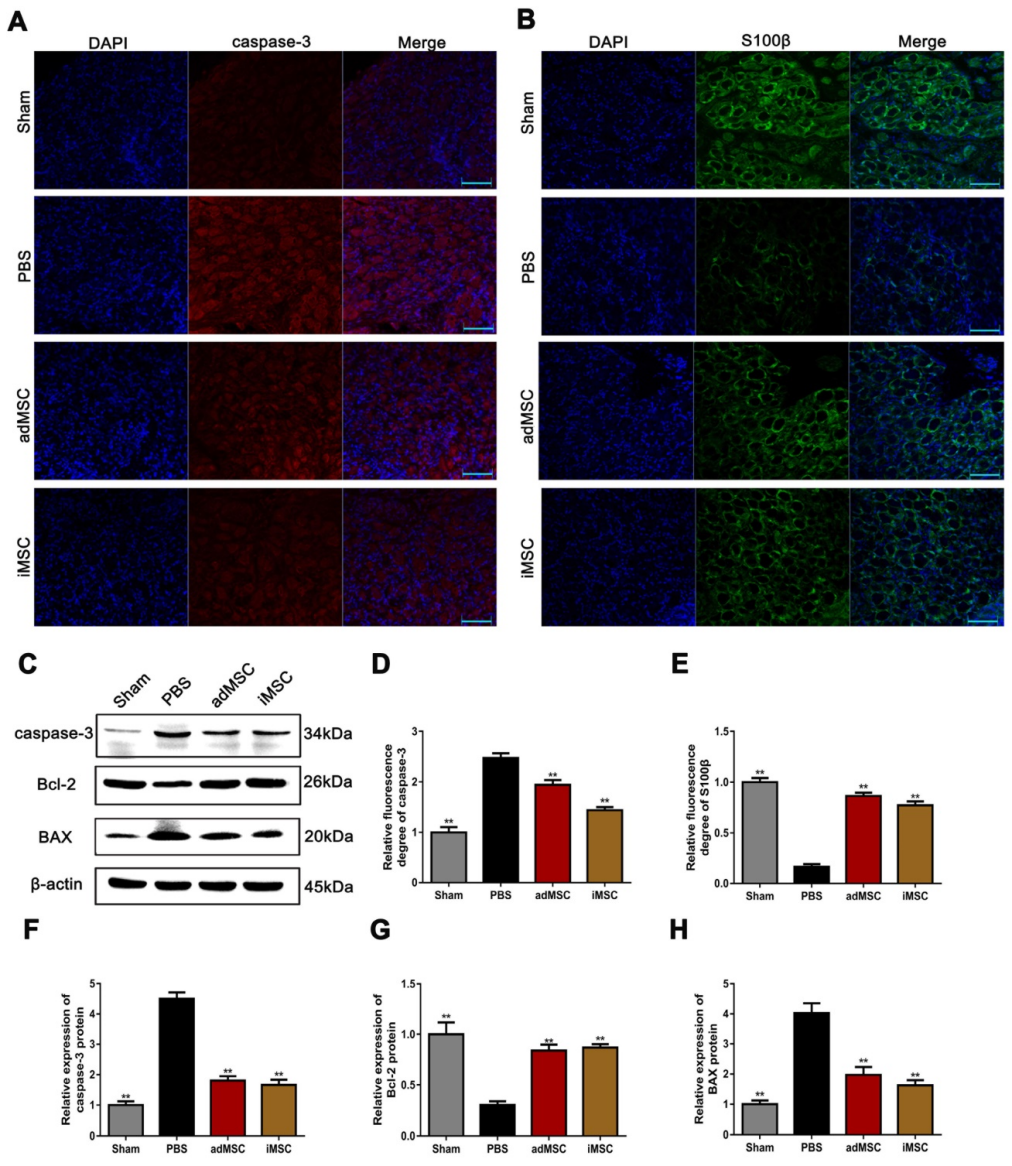
iMSC therapy exerted anti-apoptotic effects and rescued S100β expression in the MPG. (A, D) Immunofluorescent staining of caspase-3 in the MPG from the sham, PBS, adMSC, and iMSC groups. (B, E) Immunofluorescent staining of S100β in the MPG in the four groups. (C, F-H) Caspase-3, Bcl-2, and BAX protein expression in penile tissue was determined by western blot in the four groups. Error bars: mean ± SD. Bar=40 µm. **p<0.01 comparison with PBS group.

We performed immunofluorescent staining on MPG samples. The expression of the apoptosis-associated proteins caspase-3 and neural component S100β were assessed. The results showed that the CNI procedure could profoundly induce the expression of caspase-3 and iMSC or adMSC therapy could attenuate its expression (Figure 6A&D). Moreover, the intensity of S100β was remarkably weaker in the PBS group than in the sham group, but higher in the iMSC and adMSC groups (Figure 6B&E). In addition, we also detected the expression of apoptosis-related proteins (caspase-3, Bcl-2, and BAX) in the penis using western blot. CNI increased the expression of caspase-3 and BAX while repressing Bcl-2, suggesting it has pro-apoptotic effects. But these effects could be attenuated by iMSC therapy (Figure 6C&F-H). Thus, we concluded that iMSC therapy was as effective as adMSC therapy and could exert anti-apoptotic effects and rescue S100β expression in MPG.

### iMSC exerted long-term therapeutic effect and the longevity and transdifferentiation capacity were determined in nude rat

**Figure 7:**
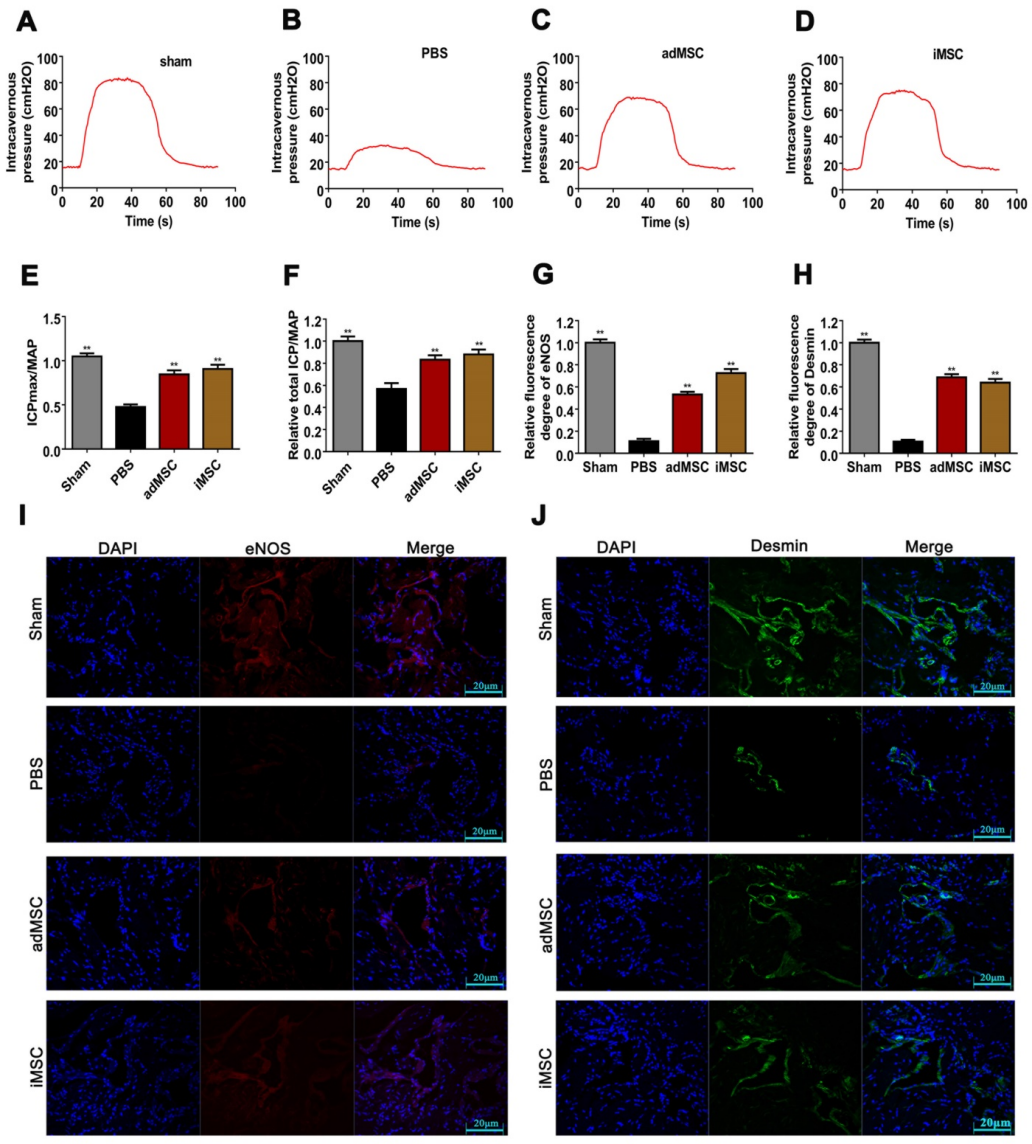
The iMSC therapeutic effect lasted 3 months in nude rat but showed poor longevity and transdifferentiation potential. (A-D) The ICP of nude rats in sham, PBS, iMSC and MSC groups were presented in three months after injection. (E) The maximum ICP to MAP ratio of nude rats were showed. (F) Total ICP to MAP ratio of nude rats were presented. (G, I) Immunofluorescent staining of eNOS in penile tissue of nude rats in three months after injection. (H, J) Immunofluorescent staining of Desmin in penile tissue of nude rats in three months after injection. Error bars: mean ± SD. **p<0.01 compare with PBS group.

**Figure 8:**
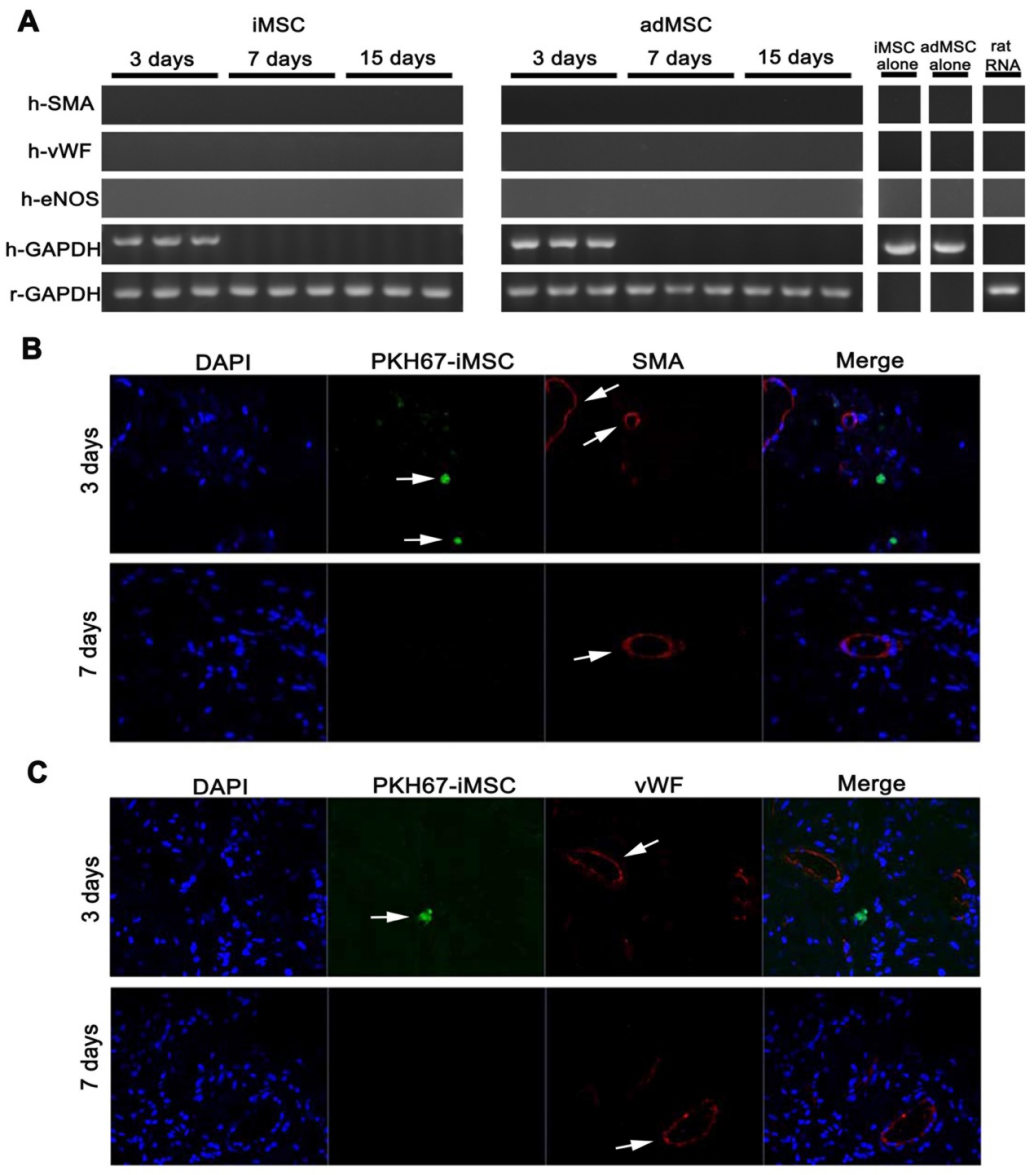
The iMSC showed poor longevity and transdifferentiation potential in nude rat ED model. (A) The transcripts for human smooth muscle (SMA) and endothelial markers (vWF and eNOS) were detected in 3, 7, and 15 days after intracavernousal injection in penis of CNI ED nude rats. The longevity of iMSC and adMSC in penis were determined by the transcripts of human GAPDH. iMSC RNA, adMSC RNA, and rat universal mRNA were also measured. Rat GAPDH was adopted as control. (B-C) There was no evidence that iMSC transdifferentiated into smooth muscle cell or endothelial cell for no PKH-67 labelled iMSC colocalized with SMA or vWF expression. The iMSC were also disappeared 7 days after intracavernousal injection in penis of CNI ED nude rats.

To further confirm the therapeutic effect, we adopted immunodeficient nude rats and established CNI ED model and made MSC injection. Three months (90 days) after MSC administration, the erectile function of CNI ED nude rats were measured. As showed, the therapeutic effect of iMSC lasted for three months and the ameliorated function was comparable to adMSC (Figure 7A-F). Moreover, the detection of eNOS, Desmin, and RECA-1 revealed that iMSC administration could obviously rescue the endothelial and smooth muscle contents of the corpus cavernosum (Figure 7G-J, Figure S2). To further understand the underlying mechanism, we labelled iMSC and adMSC with PKH67 and injected it into the cavernosum of CNI ED nude rats. The MSC longevity and its transdifferentiation capacity of specific and major cell types in cavernosum, which included smooth muscle cells and endothelial cells, were determined. Classic PCR and gel electrophoresis were performed on penis from CNI ED nude rat in 3, 7 and 15 days after MSC administration using human specific primers. As presented, the transcripts for human GAPDH were only detected in days 3 but disappeared later (Figure 8A). The monitor for PKH67 labelled iMSC revealed that iMSC could only be identifiable in first 3 days but vanished later (Figure 8B-C). These results indicated that the iMSC could only survive 3 days after injection. The transcripts for human SMA, vWF, and eNOS were not detected in both groups in all time points (Figure 8A). What's more, immunohistochemical stainings demonstrated that the iMSC labelled with PKH67 in penis did not colocalize with endothelial cell markers or smooth muscle cell markers (Figure 8B-C). The immunohistochemical stainings of adMSC showed similar results (Figure S3).These results indicated that limited iMSC transdifferentiation and there should be other mechanisms for iMSC therapeutic functions.

### iMSC exerted its function by activating a secretome in the host

**Figure 9:**
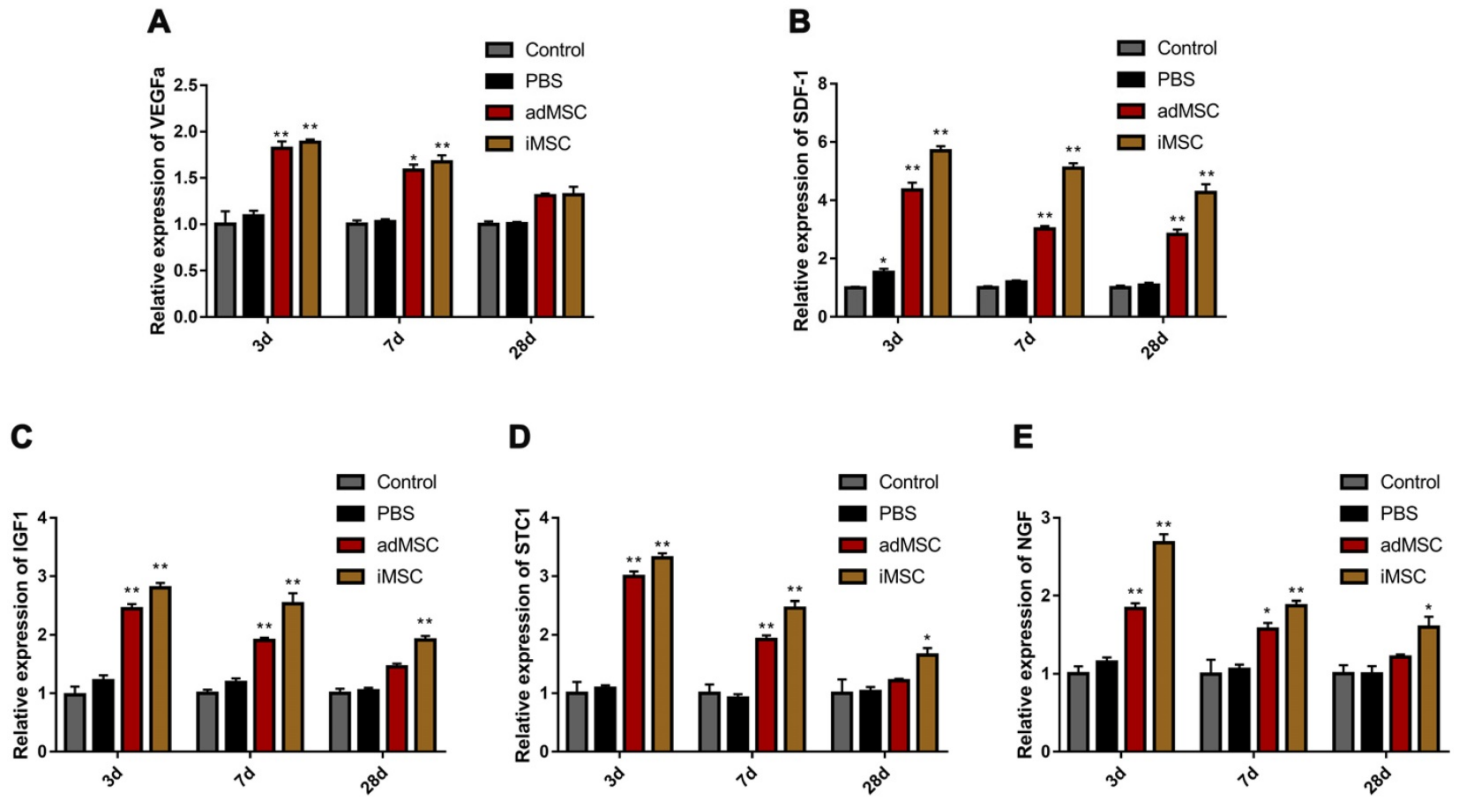
iMSC exerted their effects by activating the secretome of the host. (A-E) The expression of rat VEGFa, SDF1, STC1, IGF1, and NGF was evaluated 3, 7, and 28 days after treatment in the sham, PBS, adMSC, and iMSC groups. Error bars: mean ± SD. *p<0.05, **p<0.01 comparison with PBS group.

One of the most important mechanisms of stem cell therapy is paracrine activity. We conducted real-time PCR analysis of penile tissues collected from CNI rats that received MSC or PBS therapy. We explored the expression of secreted soluble factors that are known to affect tissue repair. These factors were vascular endothelial growth factor a (VEGFa), insulin growth factor 1(IGF1), stromal cell-derived factor 1 (SDF1), stanniocalcin 1 (STC1), and nerve growth factor (NGF).These factors are closely associated with angiogenesis, neurogenesis, anti-apoptosis, and anti-oxidative stress response. These factors were upregulated in the iMSC group at 3 days after injection. Moreover, the host secretome alteration modified by iMSC persisted over 28 days, which indicated long-term effects of iMSC administration (Figure 9A-E).

## Discussion

ED caused by pelvic surgery is a worldwide problem. It is an important complications and affects patients' psychological and physical health 28. In particular, postoperative ED is a problem in patients undergoing pelvic surgery for malignant tumors. Injuries to neurovascular bundles, and especially to nerves that are essential to sexual function, such as the CN, are sometimes unavoidable when a radical tumor resection is necessary 29. ED due to such neurovascular injuries is often not well responsive to PDE5 inhibitors or, if they are, the side effects may be intolerable. Hence, new strategies for treating this kind of ED are urgently needed 30.

Emerging evidence has revealed that MSC transplantation is effective in tissue engineering and functional repair. Some studies have shown that MSC administration is effective in alleviating ED. For example, Ryu et al. established a diabetic animal ED model and found that implantation of MSC could improve erectile function 31. You et al. performed periprostatic transplantation of mesenchymal stem cells in a CNI ED model. Their results demonstrated that administration of MSC was effective in the recovery of erectile function 32. These studies suggest that MSC is a promising tool for ED therapy. Traditionally, MSC could be derived from skeletal muscle, bone marrow, and adipose tissue 33,34. Now iPSC technology allows for new sources of MSC, call iMSC in this context. Lian et al. found that iMSC could attenuate limb ischemia in mice 23. Another study evaluated the anti-inflammatory abilities of iMSC and demonstrated that they exerted similar anti-inflammatory effects as those of adMSC in a murine model of corneal injury 24. However, to our knowledge, no studies have explored the effects of iMSC on ED.

In the present study, we first used iMSC, to treat CNI ED rat models. We introduced iMSC and evaluated their therapeutic effect on erectile function. The analysis of ICPmax/MAP and total ICP/MAP suggests that iMSC are comparable to adMSC in their effects on ED. One of the important causes of CNI ED is the deprivation of smooth muscle and endothelial tissue 7. By assaying vWF, eNOS, SMA and Desmin expression, we found that iMSC were as effective as adMSC in restoring the loss of smooth muscle and endothelial content in the penile after CNI. Moreover, administration of iMSC mitigated loss of nNOS expression which contributed to the lack of responsiveness to PDE5 inhibitors 35. We then tried to explore the underlying mechanism of how iMSC alleviated CNI ED. The nude rat CNI ED model were used for assessing the longevity and differentiation capacity of iMSC and it indicated that the cells only preserved in the penile for three days and no transdifferentiation occurred. Another important mechanism of MSC therapy is paracrine. We proved that injection of iMSC activated the host's secretion of many important soluble factors and these effects lasted long. These findings indicate that iMSC may act through a paracrine mechanism.

The development of iMSC enables new strategies in cell therapy. Traditional MSCs have obvious limitations, such as low expandability and susceptibility to senescence 36, 37. iMSC have advantages over MSC and may be a better source for biotherapy. First, iPSC can be induced from somatic cells such as skin cells, and thus they can be obtained noninvasively. Second, iMSC are highly expandable, capable of more than 120 population doublings without senescence 23. Third, MSC are more variable due to differences in donors and culture conditions, while iMSC have better homogeneity because they are derived from a single iPSC.

Our presented study showed that iMSC and adMSC appeared similar beneficial effects on recovery of erectile function and histological structure, which indicated that iMSC was also an important therapy cell for ED. Most researchers have focused on the therapeutic functions of adMSC on ED before and presented promising results. Hence, the current study also provokes an important thought that whether it is necessary to use iMSC, instead of MSC for ED. The authors hold the opinion that iMSC should be a better cell source for ED therapy for it has many advantages over adMSC and better clinical application prospect. Even not, it should be an alternative to adMSC for ED therapy in the future.

One of the specialties of our study is that we adopt a mixture of fibrin sealent and MSCs. There are two reasons why we used fibrin scaffold. The first reason is that previous study has revealed that intracavernous adMSC injection, but not perineural in cavernous, could improve erectile function 38. However, even our research team has focused on this area for a long time and skilled in establishing CNI ED model, we found it is hard to distinguish between intracavernous and perineural area in cavernous body when making injection. Hence, when making injection we mixed MSC with fibrin scaffolds, inserted the needle into the lateral cavernosum about 5 mm, withdrawed the needle slowly and made injection simultaneously. This strategy would increase the probability that the cells were injected into the cavernosum and our results indicated that it was benefit for the erectile function improvement.

Secondly, the two main hypotheses of MSC therapy for ED include repairing the crushed nerve and restoring the target organ corpus cavernosum function 9, 32. In present study, we focused on cavernosum and hypothesized that iMSC would restore the corpus cavernosum function by activating a secretome in the host. The corpus cavernosum is an organ which is rich in blood flow and if the cells were mixed with fibrin scaffold, it might not be easy to be swept away by blood flow and the therapeutic effects may last longer. However, we did not explore whether using a fibrin scaffold would influence the migration capability, viability and differentiation capacity of MSC. On future study we would step into this area and make a better understanding on the effects of fibrin scaffold.

There are some limitations to our study. Because we used a rat model, the results may not be indicative of outcomes in humans. The effects of iMSC on CNI ED in humans will require future studies. However, some studies have indicated that MSC may be tumorigenic, while one study indicated that iMSC has much less potential to promote tumors than bone marrow MSC 39. Nonetheless, trials of iMSC will require a long observation period for proper assessment of safety.

## Conclusion

To our knowledge, this study is the first to explore the function of iMSC on ED. We found that transplantation of iMSC could significantly restore erectile function induced by CNI. Injection of iMSC not only restored the IAPmax/MAP and total IAP/MAP ratio, but also counteracted the loss of endothelial and smooth muscle tissue in the penis and nNOS downregulation. We also found that iMSC may exert their benefits via paracrine effects. In sum, iMSC may be a promising therapeutic candidate for treating CNI ED in the future.

## Supplementary Material

Supplementary figures and tables.

## Abbreviations

ED: erectile dysfunction
PDE5: phosphodiesterase-5
MSC: mesenchymal stem cells
CNI: cavernous nerve injury
iPSC: Induced pluripotent stem cells
iMSC: induced pluripotent stem cell-derived mesenchymal stem cells
adMSC: adipose-derived mesenchymal stem cells
DMEM: Dulbecco's modified Eagle's medium
FBS: fetal bovine serum
PBS: phosphate-buffered saline
IAP: intracavernous pressure
MAP: mean arterial blood pressure
MPG: major pelvic ganglia
SMA: smooth muscle actin
VEGFa: vascular endothelial growth factor a
IGF1: insulin growth factor 1
SDF1: stromal cell-derived factor 1
STC1: stanniocalcin 1
NGF: nerve growth factor
