# Supplementary material for: Transplantation of induced pluripotent stem cell-derived mesenchymal stem cells improved erectile dysfunction induced by cavernous nerve injury
Source: Theranostics. 2019 Aug 14;9(22):6354–68. doi: 10.7150/thno.34008 (PMC6771238; doi:10.7150/thno.34008)
Supplement: Supplementary file 1 — Supplementary figures and tables. [file thnov09p6354s1.pdf]

Supplementary material

Table S1. Classical PCR primers

**Primer Name**	**Primer sequence (5′-3′)**	
	**Forward**	**Reverse**
human		
SMA	CCCGGGACTAAGACGGGAAT	AGCATTTGCGGTGGACAATG
vWF	CACCTGCATTTGCCGAAACA	CCGAAAGGTCCCAGGGTTAC
eNOS	AACAGCATCTCCTGCTCAGAC	CAGATTAAGGCGGACCCAGG
GAPDH	TGAATGGGCAGCCGTTAGG	TGGACTCCACGACGTACTCA
rat		
GAPDH	GTGCCAGCCTCGTGCTGATAGA	CGCCAGTAGACTCCATGACA

Table S2. Quantitative real time PCR Primers

Primer Name (rat)	Primer sequence (5′-3′)	
	**Forward**	**Reverse**
VEGFa	ACCCTGGCTTTACTGCTGTACCT	GCAATAGCTGCGCTGGTAGAC
SDF1	ATCTGAAAATCCTCAACACTCCAAA	GCACACTTGTCTGTTGTTGCTTT
STC1	TGATTCTGGCGCTGGTCATC	CTGAATTTTGAGCCGCCACC
IGF1	TGAGCGCACCTCCAATAAAGA	AACTGAAGAGCGTCCACCAG
NGF	TGCCCCTGCTGAACCAATAG	GAAGACTGGGTGGGTGGATG
GAPDH	GTTACCAGGGCTGCCTTCTC	GATGGTGATGGGTTTCCCGT

Supplementary Figure legends

Figure S1. iMSC therapy increased eNOS and Desmin expression in the penis of CNI ED rats four weeks after injection. (A, C) The expression of endothelial cell marker eNOS could be obviously rescued after iMSC therapy. (B, D) The expression of smooth muscle cell marker Desmin could be significantly ameliorated after iMSC therapy. Error bars: mean ± SD. ***p*<0.01 comparison with the PBS group.

Figure S2. iMSC therapy increased RECA-1 expression in the penis of CNI ED nude rats three months after injection. (A-B) Immunofluorescent staining on penile tissue of nude rats in three months after injection showed that the expression of RECA-1 expression was remarkably rescued by iMSC therapy. Error bars: mean ± SD. Bar=40 μm. ***p*<0.01 comparison with PBS group.

Figure S3. The adMSC showed no transdifferentiation after injection in the corpus cavernosum of nude rats. (A) The PKH67 labelled adMSC could be identified in days 3 but vanished in days 7. There was no colocalization between adMSC and SMA. (B) There was no colocalization could be detected between adMSC and vWF.

Figure S1.

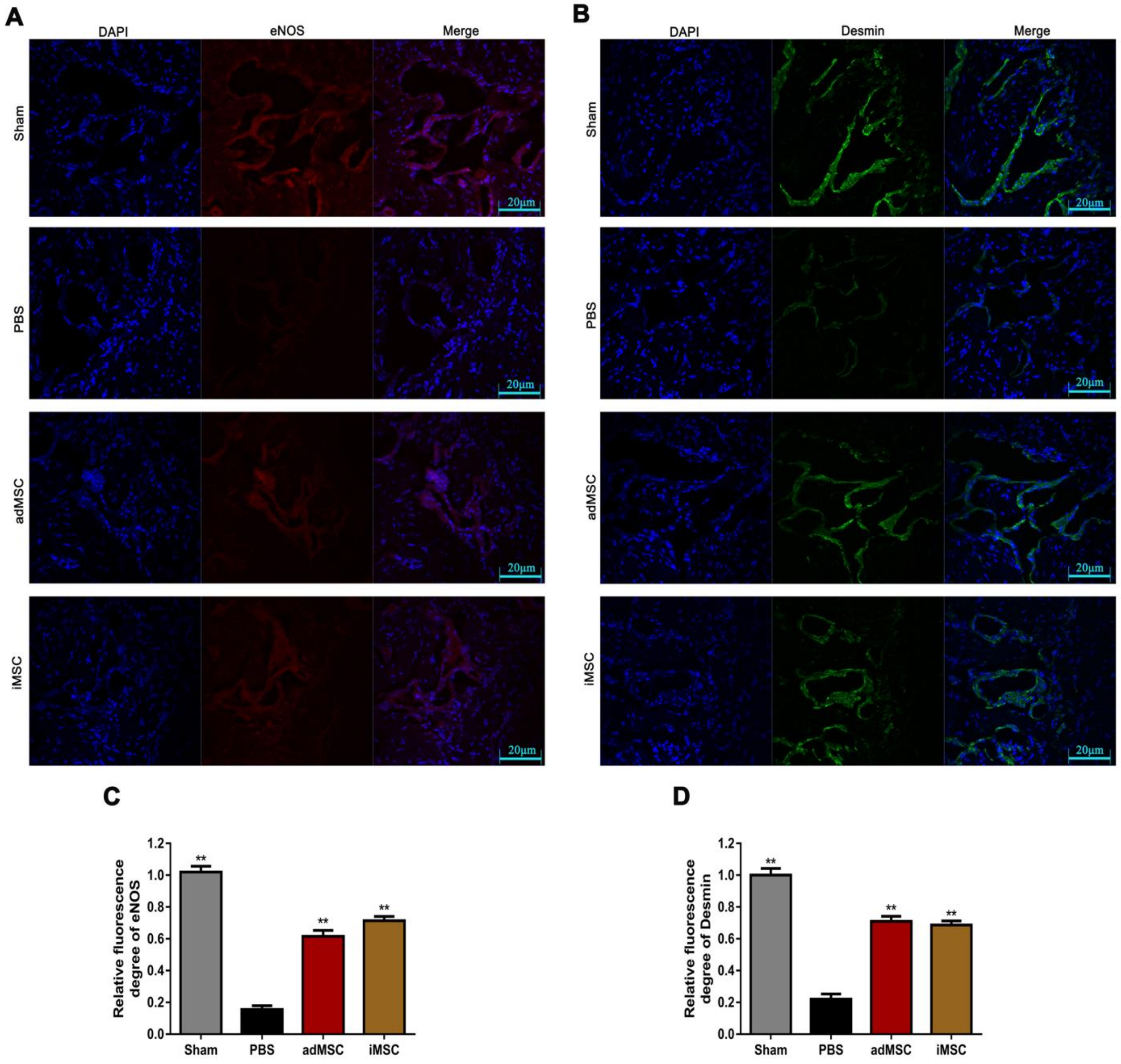
A
DAPI
eNOS
Merge
Sham
PBS
adMSC
iMSC
20μm
B
DAPI
Desmin
Merge
C
Relative fluorescence degree of eNOS
1.2
1.0
0.8
0.6
0.4
0.2
0.0
**
Sham
PBS
adMSC
iMSC
D
Relative fluorescence degree of Desmin

Figure S2.

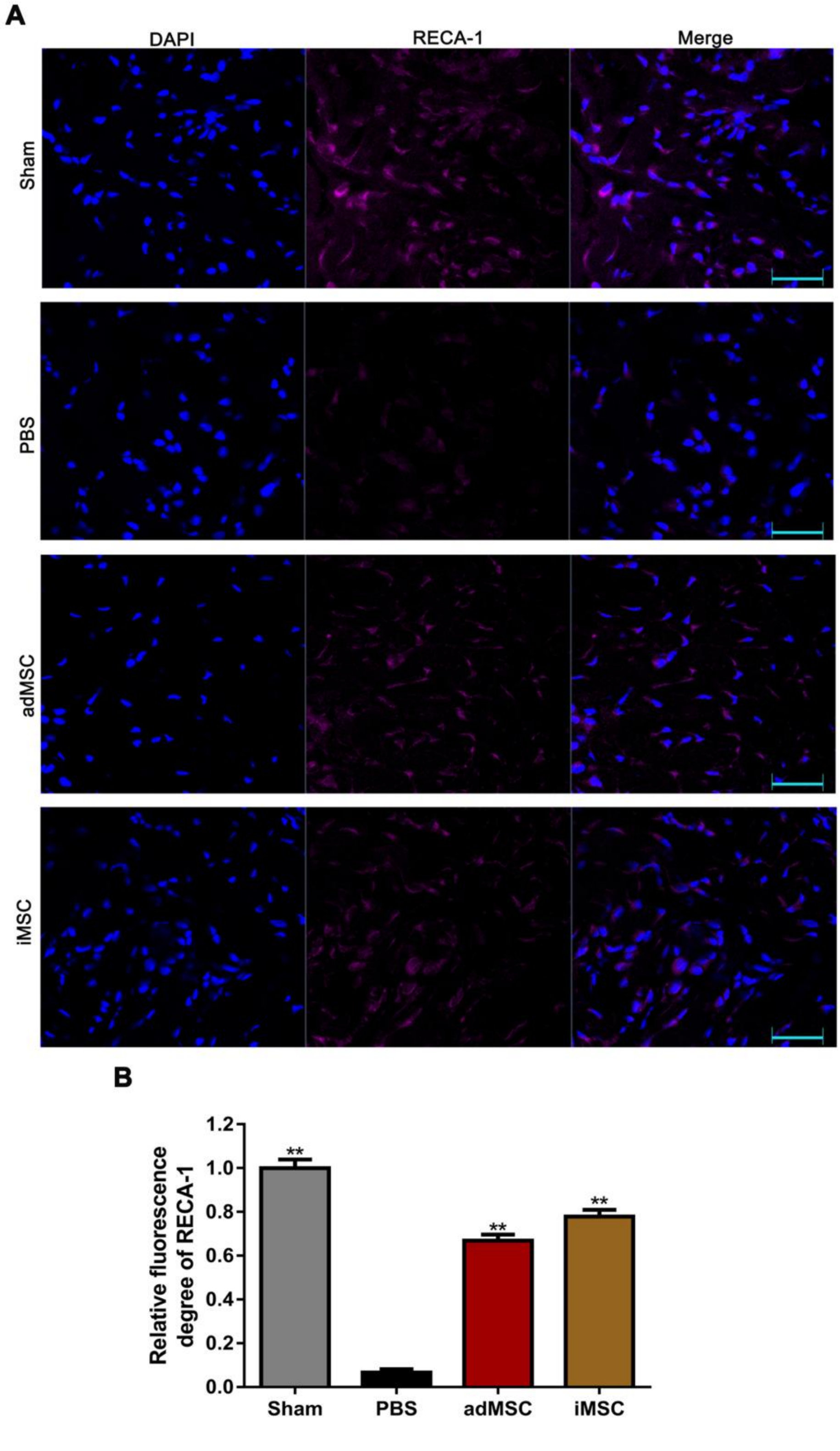
A
DAPI
RECA-1
Merge
Sham
PBS
adMSC
iMSC
B
Relative fluorescence degree of RECA-1
0.0
0.2
0.4
0.6
0.8
1.0
1.2
**
**
**
Sham
PBS
adMSC
iMSC

Figure S3.

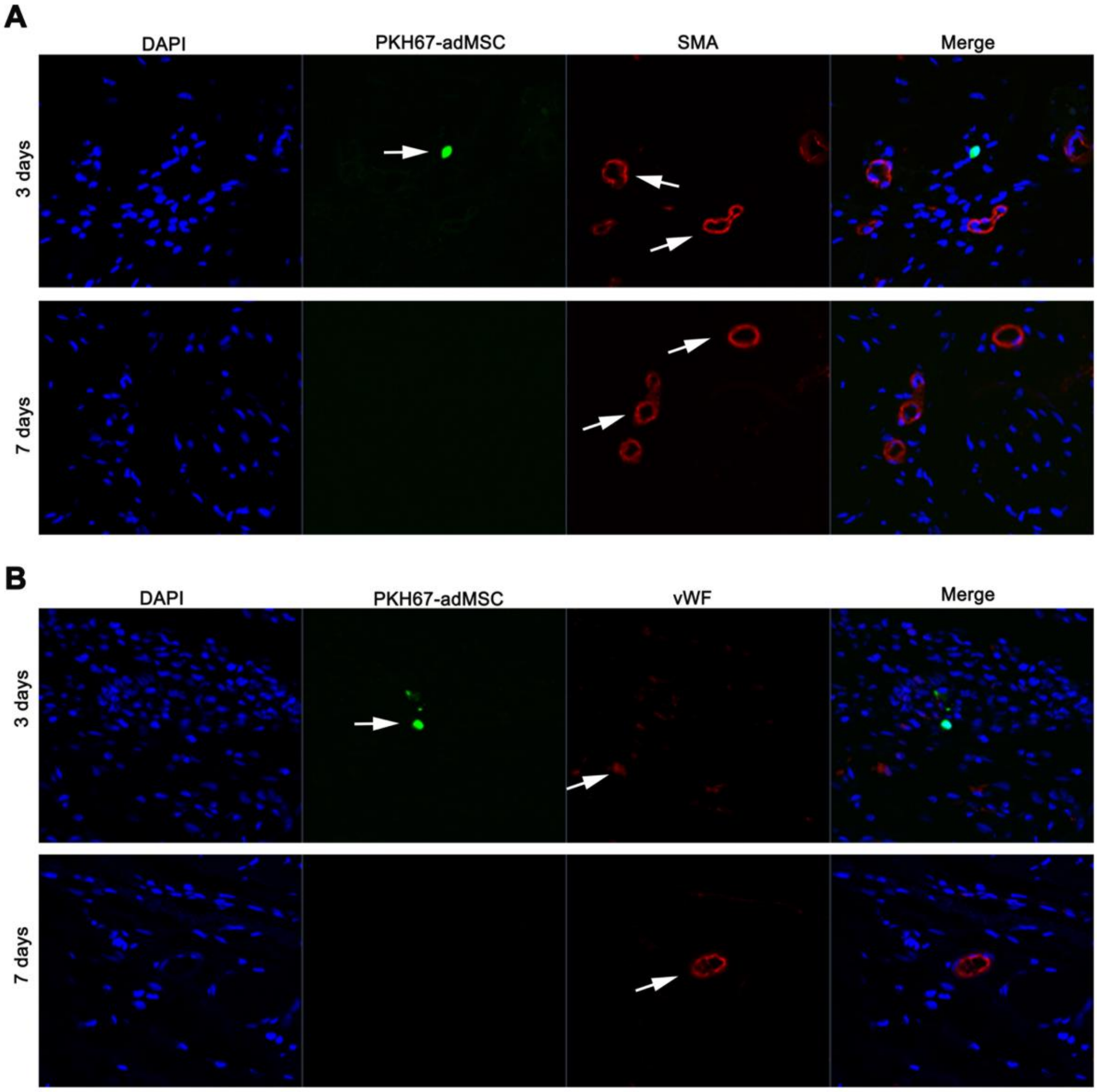
A
DAPI
PKH67-adMSC
SMA
Merge
3 days
7 days
B
DAPI
PKH67-adMSC
vWF
Merge
3 days
7 days
